# Assessing CIAS in clinical routine

**DOI:** 10.1192/j.eurpsy.2025.211

**Published:** 2025-08-26

**Authors:** A. Erfurth, G. Sachs

**Affiliations:** 1 1st Department of Psychiatry and Psychotherapeutic Medicine, Klinik Hietzing; 2 Medical University of Vienna, Vienna, Austria

## Abstract

**Abstract:**

Cognitive impairment associated with schizophrenia (CIAS) is a relevant problem and can be well assessed in different ways, as described in an EPA guidance paper (1). In routine clinical practice, the clinician usually relies on targeted questions to elicit psychopathological findings, as described in the AMDP system (2), for example. If there is a need for a more precise assessment of cognitive dysfunction, also in a transdiagnostic way, the Screen for Cognitive Impairment in Psychiatry (SCIP) (3) has recently been used by several working groups.

In a recent pilot study (4), we used the German version of the SCIP (SCIP-G) (5,6) to assess cognitive impairment in acute psychiatric inpatients diagnosed with psychotic disorders, bipolar disorder and depression.

(1) Vita A, Gaebel W, Mucci A, Sachs G, Erfurth A, Barlati S, Zanca F, Giordano GM, Birkedal Glenthøj L, Nordentoft M, Galderisi S. European Psychiatric Association guidance on assessment of cognitive impairment in schizophrenia. Eur Psychiatry. 2022 Sep 5;65(1):e58. doi: 10.1192/j.eurpsy.2022.2316.

(2) The AMDP System: Manual for Assessment and Documentation of Psychopathology in Psychiatry, 9th edition. 2017. Ed.: Broome MR, Bottlender R, Rösler M, Michael; Stieglitz RD. Hogrefe Publishing GmbH. ISBN: 978-0-88-937542-0

(3) Purdon SE. The Screen for Cognitive Impairment in Psychiatry (SCIP): Instructions and three alternate forms. 2005. PNL Inc, Edmonton, Alberta

(4) Maihofer EIJ, Sachs G, Erfurth A. Cognitive Function in Patients with Psychotic and Affective Disorders: Effects of Combining Pharmacotherapy with Cognitive Remediation. J Clin Med. 2024 Aug 16;13(16):4843. doi: 10.3390/jcm13164843.

(5) Sachs G, Lasser I, Purdon SE, Erfurth A. Screening for cognitive impairment in schizophrenia: Psychometric properties of the German version of the Screen for Cognitive Impairment in Psychiatry (SCIP-G). Schizophr Res Cogn. 2021 May 12;25:100197. doi: 10.1016/j.scog.2021.100197.

(6) Sachs G, Bannick G, Maihofer EIJ, Voracek M, Purdon SE, Erfurth A. Dimensionality analysis of the German version of the Screen for Cognitive Impairment in Psychiatry (SCIP-G). Schizophr Res Cogn. 2022 Jun 6;29:100259. doi: 10.1016/j.scog.2022.100259.

**Disclosure of Interest:**

None Declared

